# The Efficacy of High-Dose Chemotherapy Followed by Autologous Stem Cell Transplantation in Ewing Sarcoma Patients

**DOI:** 10.3390/jcm14248621

**Published:** 2025-12-05

**Authors:** Ömer Faruk Kuzu, Nuri Karadurmuş, Nebi Batuhan Kanat, Çağlar Köseoğlu, Ayşegül Dumludağ, Alper Topal, Ramazan Acar, Birol Yıldız, Musa Barış Aykan, İsmail Ertürk

**Affiliations:** 1 Oncology, Cankiri State Hospital, Çankırı 18100, Turkey; 2 Division of Medical Oncology, Department of Internal Medicine, Gulhane Research & Training Hospital, Ankara 06010, Turkey; drnkaradurmus@yahoo.com (N.K.); musabarisaykan@gmail.com (M.B.A.); ierturk@hotmail.com (İ.E.); 3 Division of Medical Internal Medicine, Department of Internal Medicine, Gulhane Research & Training Hospital, Ankara 06010, Turkey; nebibatuhan.kanat@sbu.edu.tr; 4 Oncology, Gaziantep City Hospital, Gaziantep 27470, Turkey; drcaglarkoseoglu@hotmail.com; 5 Oncology, Erzurum Regional Education and Research Hospital, Erzurum 25240, Turkey; dr.aysegulcomakli@gmail.com; 6 Oncology, Tokat Gaziosmanpasa University, Tokat 60030, Turkey; dralpertopal@gmail.com; 7 Oncology, Ufuk University Dr Rıdvan Ege Hospital, Ankara 06510, Turkey; dr_racar@yahoo.com (R.A.); bfyildiz@gmail.com (B.Y.)

**Keywords:** Ewing sarcoma, high-dose chemotherapy, autologous stem cell transplantation, prognostic factors

## Abstract

**Background:** Ewing sarcoma (ES) is a highly aggressive malignant tumor that predominantly affects children and young adults. Despite advances in multimodal therapy, relapse and refractory disease remain the leading causes of treatment failure. High-dose chemotherapy followed by autologous stem cell transplantation (HDCT-ASCT) has been proposed as a consolidation strategy for high-risk or relapsed ES; however, its clinical value remains controversial. **Methods:** We retrospectively analyzed 46 consecutive patients with locally advanced or metastatic ES who underwent HDCT-ASCT after at least one prior systemic therapy line. Clinical, pathological, and transplant-related variables were evaluated for associations with overall survival (OS), post-transplant OS (OS-2), and progression-free survival (PFS). Survival was estimated using the Kaplan–Meier method, and prognostic factors were assessed by Cox proportional hazards modeling. **Results:** The median age at diagnosis was 23 years (range: 14–55). Median OS from diagnosis was 42 months, while post-transplant OS-2 and PFS were 8 and 5 months, respectively. Younger patients (≤23 years) had significantly longer OS (50 vs. 34 months; *p* = 0.027). Liver metastasis predicted inferior OS (HR = 5.411; *p* = 0.006), whereas lung metastasis was associated with shorter OS-2 (HR = 2.672; *p* = 0.025) and PFS (HR = 6.037; *p* = 0.016). Treatment-related mortality was low (2.1%), though hematologic toxicity was universal. Overall, HDCT-ASCT provided transient disease control, with modest benefit confined to younger, chemosensitive, and medically fit patients. **Conclusions:** In this real-world cohort, HDCT-ASCT was feasible and safe but offered limited survival advantage in heavily pretreated Ewing sarcoma. Prognosis was primarily influenced by age and metastatic distribution, particularly hepatic and pulmonary involvement. These findings support a risk-adapted, biology-driven approach reserving HDCT-ASCT for selected patients and highlight the need for post-transplant maintenance strategies integrating targeted or immunotherapeutic modalities.

## 1. Introduction

Ewing sarcoma (ES) is the second most common primary bone malignancy in children and adolescents after osteosarcoma [1,2]. Despite decades of collaborative progress integrating multi-agent chemotherapy, surgery or radiotherapy for local control, and risk-adapted stratification, survival outcomes have plateaued. Approximately one-quarter of patients present with metastatic disease at diagnosis, and long-term outcomes remain poor: disease-related mortality occurs in roughly 30–40% of patients with localized ES and up to 80% among those with metastatic involvement [3].

The introduction of interval-compressed vincristine–doxorubicin–cyclophosphamide alternating with ifosfamide–etoposide (VDC/IE) has modestly improved event-free survival (EFS) in both localized and metastatic settings [4,5]. Nevertheless, relapse remains the major barrier to cure—most frequently occurring within the first two years, although late recurrences are not uncommon [6,7,8]. Once relapse happens, the prognosis is grim and highlights the critical need for novel consolidation strategies capable of achieving durable remission.

High-dose chemotherapy with autologous stem cell transplantation (HDCT-ASCT) has been investigated for several decades as a means of eradicating residual tumor cells and achieving longer survival in patients with high-risk, relapsed/refractory ES. Indeed, after several years of study its actual therapeutic potential remains unknown. Feasibility of dose intensification with an alkylating agent in patients with ES over several chemotherapy blocks has been supported by some large prospective cooperative trials, such as the Euro-EWING 99 and EWING 2008, which have unfortunately shown no sustained improvement in overall survival (OS) or event-free survival (EFS), but a significantly increased treatment-related toxicity [9,10]. On the other hand, several retrospective multi-center analyses have reported somewhat more favorable albeit variable findings which suggest that at least a subset of patients—more notably those with chemosensitive disease, made it to second complete remission (CR2) or had a protracted DFI—may derive meaningful benefit [11,12,13,14].

This persistent gulf between the results of controlled trial experience and real-world clinical practice challenges an important question which endures: who are the patients most likely to gain benefit from HDCT-ASCT, and in what context biological or clinical? It is important to do this, as this therapy is invasive, expensive and with potential morbidity but also of a possibility for long-lasting remission in certain responding patients.

Given these unknowns, we conducted a single-center retrospective study to evaluate real-world outcomes in patients with advanced or relapsed ES who received HDCT-ASCT following at least one prior line of systemic therapy. The primary outcomes were PFS, overall survival post-transplant (OS-2) and OS. We also investigated clinicopathologic variables (age, primary tumor site, metastatic organ) as prognostic factors. This study will correlate these outcomes of survival with more specific clinical characteristics, not only in order to bring this therapy more into focus but also to begin developing a much-needed evidence-based context in which we might couple (or de-couple) its use according to certain risk factors in each advanced or relapsed Ewing sarcoma.

## 2. Materials and Methods

### 2.1. Study Design and Setting

We retrospectively analyzed a single-center cohort of patients with histologically proven Ewing sarcoma who received high-dose chemotherapy and HDCT-ASCT. We included 46 consecutive eligible patients with both baseline and follow-up data available.

All patients with pathologically confirmed Ewing sarcoma who underwent HDCT-ASCT at our center between January 2017 and December 2024 were evaluated. In accordance with institutional practice, only patients who had received at least one prior line of systemic therapy were considered eligible for high-dose chemotherapy. Patients with ECOG performance status 3–4 were not candidates for HDCT-ASCT because such patients are unable to safely tolerate high-dose therapy; therefore, they were not included in the study population. Follow-up continued until death for deceased patients, whereas survival endpoints for living patients were censored at their last documented clinical visit. Thus, OS, OS-2, and PFS were calculated using either the date of death or the last known follow-up date, whichever occurred first.

### 2.2. Eligibility Criteria

Eligible patients were those who fulfilled the following; histologically proven Ewing’s sarcoma, standard HDCT-ASCT as per institutional guidelines at any of the included centers and adequate clinical and follow-up data available for studying survival end points. Patients with missing important information or poor follow-up were excluded.

### 2.3. Data Collection and Variables

Demographics, clinical notes, and treatment details were retrieved from electronic health records. Collected parameters were age at diagnosis, sex, disease stage (locally advanced or metastatic), primary tumour site (upper limb, lower limb, vertebrae and pelvis or soft tissue) and baseline metastatic sites (lung, liver, bone and brain) as binary variables. The number of previous systemic therapy lines before HDCT-ASCT (≤2 vs. >2) was also recorded. Transplant-related variables were stem-cell yield and manifestations of haematologic recovery.

### 2.4. Transplantation Procedure and Definitions

All ASCT recipients underwent a high-dose ICE (ifosfamide, carboplatin, etoposide) conditioning regimen according to institutional practice. In patients who received ICE as the HDCT protocol, the total dose consisted of 12 g/m^2^ ifosfamide with mesna, 1200 mg/m^2^ carboplatin, and 1200 mg/m^2^ etoposide, administered over six consecutive days (days −8, −7, −6, −5, −4, and −3), followed by autologous stem cell infusion on day 0. High-dose chemotherapy was typically initiated 3–6 weeks after completion of the last systemic therapy cycle, depending on hematologic recovery, performance status, and transplant readiness. Supportive care measures followed institutional guidelines, although minor variations in supportive measures occurred due to ancillary factors. Engraftment was defined as the first of three consecutive days with an ANC > 2000/µL or a platelet count > 20,000/µL without transfusion or G-CSF support. The time to engraftment was calculated as the number of days between transplantation and this first qualifying day. The cumulative pre-ASCT stem-cell dose was also recorded.

### 2.5. Three Time-to-Event Outcomes Were Evaluated

(1) Overall survival (OS): Defined as the time from diagnosis to death from any cause; surviving patients were censored at the date of their last documented follow-up.
(2) Post-transplant overall survival (OS-2): Defined as the interval between ASCT and death from any cause.
(3) Progression-free survival (PFS): Defined as the time from ASCT to radiologic progression, relapse, or death—whichever occurred first; patients without an event were censored at the date of their most recent disease assessment.

Radiologic response categories—complete response (CR), partial response (PR), stable disease (SD), and progressive disease (PD)—were determined according to RECIST 1.1 criteria using PET-CT or contrast-enhanced conventional CT. Response assessments were routinely performed approximately 3 months after HDCT-ASCT, in line with institutional practice.

### 2.6. Statistical Analysis

Baseline characteristics were summarized as counts and percentages or medians with ranges. Survival curves were estimated using the Kaplan–Meier method and compared by log-rank tests. To evaluate prognostic factors, Cox proportional hazards models were fitted for each endpoint. For models assessing primary tumor site, the upper extremity served as the reference category; for models assessing metastatic site, each organ site (lung, liver, bone, brain) was entered as a binary covariate. The proportional-hazards assumption was assessed by graphical methods based on log–log survival plots. Correlations among age at diagnosis, collected stem-cell count, and recovery time were examined using Spearman’s rank correlation (one-tailed *p*-values reflecting directional hypotheses). All other tests were two-sided with α = 0.05. Analyses were performed using IBM SPSS Statistics version 27.

## 3. Results

Among 46 patients, the median age at diagnosis was 23.5 years (range, 14–55); 32 (69.6%) were male. At presentation, 36 (78.3%) had locally advanced and 10 (21.7%) had metastatic disease. Primary sites were soft tissue in 16 (34.8%), lower extremity in 13 (28.3%), vertebra in 7 (15.2%), and pelvis and upper extremity in 5 (10.9%) each. Baseline metastases most frequently involved the lung (35; 76.1%) and bone (30; 65.2%), with less frequent liver (5; 10.9%) and brain (3; 6.5%) involvement. Before HDCT-ASCT, 37 (80.4%) patients had received two or fewer prior lines of therapy (Table 1).

**Figure 1:**
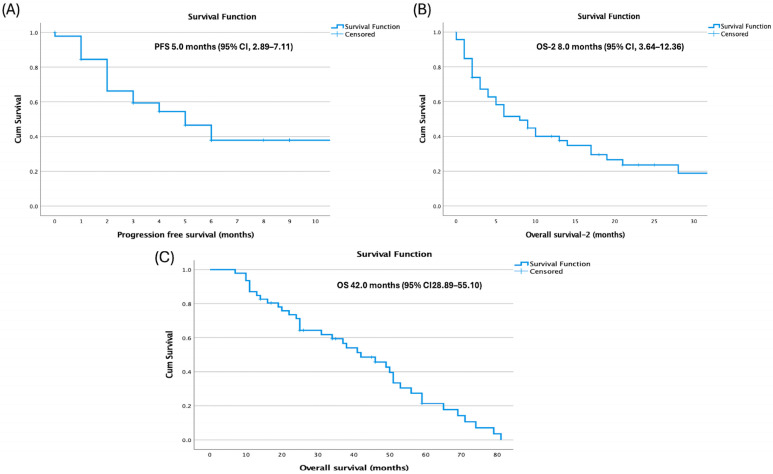
Kaplan–Meier Survival Curves (**A**) Progression free survival, (**B**) Post-transplant overall survival (OS-2), and (**C**) Overall survival from diagnosis.

Median overall survival (OS) from diagnosis was 42.0 months (95% confidence interval [CI], 28.89–55.10), median progression-free survival (PFS) after HDCT–ASCT was 5.0 months (95% CI, 2.89–7.11), and post-transplant overall survival (OS-2) was 8.0 months (Figure 1).

**Figure 2:**
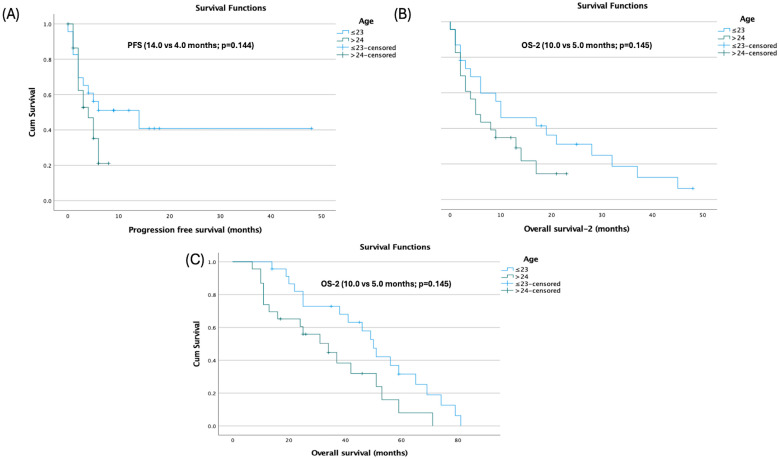
Age-stratified Kaplan–Meier survival curves: (**A**) Progression-free survival, (**B**) Overall survival-2 (OS-2), and (**C**) Overall survival.

Age at diagnosis showed differences across endpoints: patients aged ≤23 years had longer PFS (14.0 vs. 4.0 months; *p* = 0.144) and OS-2 (10.0 vs. 5.0 months; *p* = 0.145) without statistical significance, but significantly longer OS (50.0 vs. 34.0 months; 95% CI: 43.11–56.89 vs. 16.70–51.30; *p* = 0.027) (Figure 2).

**Figure 3:**
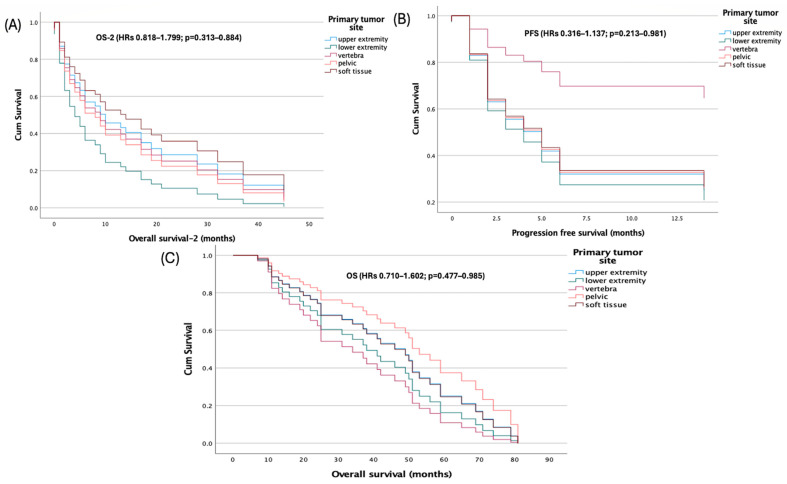
Kaplan–Meier survival curves stratified by primary tumor site. (**A**) Overall survival-2, (**B**) progression-free survival, and (**C**) overall survival.

Primary tumor site was not associated with survival in multivariable Cox models using the upper extremity as reference. Hazard ratios for lower extremity, vertebra, pelvis, and soft tissue were near unity with non-significant *p*-values for PFS (HRs 0.316–1.137; *p* = 0.213–0.981), OS (HRs 0.710–1.602; *p* = 0.477–0.985), and OS-2 (HRs 0.818–1.799; *p* = 0.313–0.884) (Figure 3).

By contrast, metastatic site showed endpoint-specific effects. For OS, the model was significant overall (score χ^2^ = 12.182, df = 4, *p* = 0.016), and liver metastasis independently predicted worse survival (HR = 5.41, 95% CI: 1.610–18.182; *p* = 0.006), whereas lung, bone, and brain metastases were not significant. For OS-2, the overall model was not significant (χ^2^ = 6.779, *p* = 0.148), but lung metastasis was associated with increased risk (HR = 2.672, 95% CI: 1.131–6.311; *p* = 0.025). For PFS, the score test approached significance (χ^2^ = 9.409, *p* = 0.052) and the change-from-block test was significant (χ^2^ = 11.078, *p* = 0.026); lung metastasis was the only significant covariate (HR = 6.037, 95% CI: 1.390–26.230; *p* = 0.016), while other sites were non-significant (Table 2).

**Figure 4:**
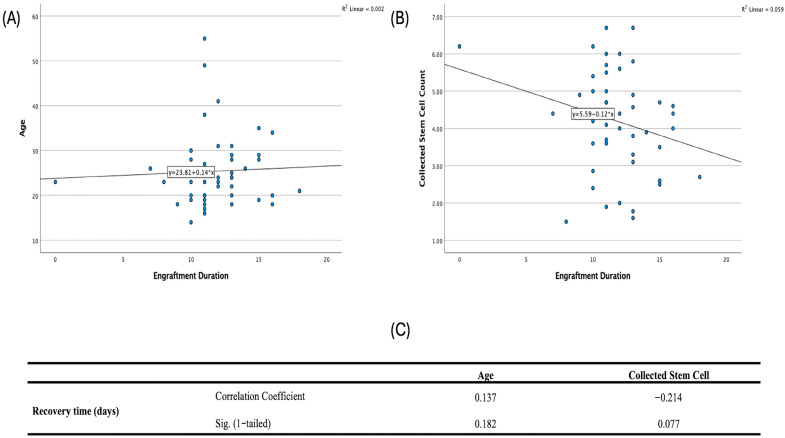
Engraftment duration versus (**A**) age, (**B**) collected stem cell count with linear trend lines, (**C**) presents the summary of Spearman correlations and one-tailed significance.

Spearman correlations showed no significant relationships among age at diagnosis, collected stem-cell count, and recovery time: age vs. stem cells (ρ = −0.056, *p* = 0.355), age vs. recovery (ρ = 0.137, *p* = 0.182), and stem cells vs. recovery (ρ = −0.214, *p* = 0.077) (Figure 4).

### Safety

Hematologic toxicities were universal, with febrile neutropenia, neutropenia, and thrombocytopenia observed in 100% of patients. Neutropenia consisted of 21.7% grade 1–2 events and 78.3% grade 3–4 events, while all thrombocytopenia cases were grade 3–4. Anemia occurred in 86.9% of patients, including 32.6% grade 1–2 and 54.3% grade 3–4 toxicities. Mucositis/stomatitis was reported in 65.2% of patients (56.5% grade 1–2; 8.7% grade 3–4). Vomiting occurred in 82.6% (17.4% grade 1–2; 65.2% grade 3–4), and diarrhea in 78.2% (11.1% grade 1–2; 88.9% grade 3–4). Liver toxicity was recorded in 21.7% of patients, all grade 1–2, while renal toxicity occurred in 17.3%, also exclusively grade 1–2. Despite the high incidence of both hematologic and non-hematologic toxicities, treatment-related mortality was low, with only one patient (2.1%) dying from complications related to HDCT-ASCT (Table 3).

## 4. Discussion

ES is a rare and biologically aggressive malignancy that predominantly affects children and adolescents. In our cohort, the median age was 23 years, representing a young adult population. Despite therapeutic progress, outcomes—particularly among patients with metastatic disease—remain suboptimal. We conducted a single-center, retrospective analysis of 46 patients with locally advanced or metastatic ES, all of whom had received at least one prior line of systemic therapy before undergoing HDCT-ASCT. The primary objective was to evaluate survival endpoints and assess clinicopathologic factors associated with OS and PFS. Accordingly, our study population predominantly represents a heavily pretreated, real-world cohort, more accurately reflecting contemporary clinical practice rather than treatment-naive trial settings.

Given that all patients had previously received chemotherapy and in many cases, multimodal local treatment, the outcomes observed in our study primarily characterize a population with potential cumulative toxicity and reduced chemosensitivity. This context is essential when interpreting survival outcomes, as prior exposure to intensive regimens is known to compromise marrow reserve and diminish responsiveness to subsequent high-dose therapy—a finding consistent with previously reported series in relapsed or refractory ES [11,12,13,14].

Based on the results of the Euro-EWING 99 and EWING 2008 trials (≈287 patients across relevant cohorts), HDCT-ASCT did not significantly improve survival among patients presenting with isolated pulmonary metastases and was associated with increased acute toxicity. Consequently, this approach has not been adopted as a routine standard for this subgroup [9]. These pivotal multicenter phase III trials compared high-dose busulfan–melphalan (BuMel) chemotherapy followed by ASCT with standard vincristine–actinomycin D–ifosfamide (VAI) chemotherapy plus whole-lung irradiation (WLI). Among the 287 patients enrolled, 143 received VAI + WLI and 144 received BuMel. The 3-year EFS was 56.6% in the BuMel arm versus 50.6% in the VAI + WLI arm, with no statistically significant difference (HR: 0.79; *p* = 0.16). Similarly, the 8-year EFS rates (52.9% vs. 43.1%) showed only a non-significant trend favoring BuMel, while OS was identical between groups (HR: 1.00; *p* = 0.99). Importantly, BuMel treatment was associated with higher acute toxicity and four treatment-related deaths. Subgroup analysis revealed a modest, non-significant trend toward improved EFS in patients without pleural involvement. By contrast, in localized high-risk ES, BuMel-based HDCT-ASCT demonstrated a clinically meaningful improvement in both EFS and OS compared with standard consolidation therapy, albeit at the cost of increased toxicity. Therefore, this approach is currently considered an option only for carefully selected, medically fit patients treated in specialized centers rather than as a universal standard therapy [9]. Similarly, in the EWING 2008 R3 trial (n = 109), high-dose treosulfan–melphalan followed by ASCT did not result in a significant improvement in EFS or OS and was associated with increased acute toxicity, although a prespecified subgroup analysis suggested a potential 3-year EFS advantage among patients younger than 14 years [10].

Consistent with these large-scale cooperative trials, our findings highlight the limited curative potential of HDCT-ASCT in advanced or metastatic Ewing sarcoma, particularly in patients previously exposed to systemic therapy. Among 46 consecutively treated patients, all of whom had received at least one prior line of systemic therapy, the median OS from diagnosis was 42 months, whereas OS-2 and PFS were only 8 and 5 months, respectively. Although younger age (≤23 years) was significantly associated with longer OS (50 vs. 34 months; *p* = 0.027), neither the primary tumor site nor the number of prior therapy lines independently influenced survival outcomes.

Importantly, the metastatic pattern exerted the strongest prognostic impact: liver metastasis predicted inferior OS (HR = 5.411; *p* = 0.006), whereas lung metastasis significantly affected both OS-2 (HR = 2.672; *p* = 0.025) and PFS (HR = 6.037; *p* = 0.016). These findings are consistent with Euro-EWING data, reaffirming that pulmonary involvement remains a critical determinant of poor prognosis even after intensive therapy.

Despite universal hematologic toxicity—including febrile neutropenia and thrombocytopenia observed in all patients—treatment-related mortality remained low (2.1%), indicating that the regimen was feasible but offered only transient disease control. The modest radiologic response rate (complete or partial response in 26.1% of patients) and the predominance of progressive disease (58.7%) likely reflect the biological aggressiveness of Ewing sarcoma following multiple prior treatments, where cumulative chemoresistance represents a major limiting factor.

These findings taken together substantiate that age and metastatic organ distribution, especially hepatic and pulmonary involvement, could aid the risk-adapted selection of HDCT-ASCT for patients with ES. Furthermore, our data highlight the need for consolidation or maintenance approaches with targeted agents, immune therapy, or molecularly directed agents to extend disease control post-transplantation in this highly treated population.

Our findings were contextualized by directly comparing them with recent randomized evidence, including the Euro-EWING 99 and EWING 2008 trials, where high-dose therapy failed to demonstrate a significant survival advantage over standard regimens despite increased toxicity. In contrast, the markedly shorter OS-2 and PFS observed in our real-world cohort likely reflect substantial differences in patient characteristics: our population consisted predominantly of young adults rather than pediatric patients, involved more heavily pretreated and clinically heterogeneous cases, and included a high proportion of patients with visceral metastases or chemoresistant disease at the time of HDCT-ASCT. These factors—together with cumulative toxicity, reduced bone marrow reserve, and the absence of stringent trial-level eligibility criteria—explain why real-world outcomes diverge from controlled trial results and highlight the need for selective, biology-driven use of HDCT-ASCT

For patients with relapsed or refractory ES, HDCT-ASCT has been examined in a number of retrospective series which yield diverse but sometimes encouraging results relative to salvage chemotherapy [11,12,13,14]. In their multicenter study, Rasper et al. in 239 patients with first-relapsed ES, HDCT–ASCT consolidation resulted in a significant 2-year EFS benefit for chemosensitive patients [11]. However, those who relapsed early (within 2 years of diagnosis) derived little benefit and these results underline that continued poor outcome is largely due to intrinsic tumour biology and chemo-resistance. These results are in line with our data, by which patients considered to have a high tumor burden experience poor OS (HR = 5.411; *p* = 0.006) despite having been transplanted, meaning that tumor biology is often the dominate determinant of therapy response over level of intensity of our systemic agents.

Likewise, Windsor et al. analyzed 196 refractory or relapsed ES cases and observed remarkable survival variation on the basis of treatment: median OS was 76 months for the HDCT-ASCT group, in contrast with 10.5 months when treated by conventional-dose strategies [12]. Significantly, the benefit itself was limited to patients who responded to induction prior to transplant and thereby emphasizes the importance of pre-transplant chemosensitivity as an important predictor. We found radiologic remission in only 26.1% of our series and progression in 58.7% just after HDCT-ASCT, which emphasizes that ultimately the potential for intensification is restricted by chemoresistance.

Similarly, Ferrari et al. recognized that disease status at transplant was the strongest independent predictor of post-relapse outcome with patients attaining a CR2 before HDCT–ASCT showing the best OS [13]. We also have had similar experience whereby patients with stable/partially responsive disease pre-transplant seemed to have a longer survival post-transplant as compared to those who were transplanted while in active progression, although this difference was not statistically significant. Taken together, these datasets suggest that the depth of remission and the optimization of timing are more critical determinants of survival than the conditioning regimen itself.

Shankar et al. also found that a disease-free interval more than 24 months was the only independent variable for better post-recurrence survival [14]. Late-relapse patients, usually indicative of less aggressive tumor biology, had much better outcomes irrespective from the type of treatment. Consistently, the previously reported young age (≤23 years; likely reflecting greater tolerance and less genomic instability) was significantly correlated with prolonged OS in our series (50 vs. 34 months; *p* = 0.027), further reinforcing that not only host characteristics but also tumor kinetics contribute to treatment response.

Collectively, these studies [11,12,13,14], as well as our results, represent a consistent conclusion: HDCT-ASCT significantly benefits only a subgroup of biologically and clinically favorable patients—those who have chemosensitive disease, less metastasis involvement, longer remission duration time without disease symptoms and good baseline condition. By comparison, patients who have early relapse, chemoresistant progression or visceral metastases (particularly hepatic involvement) derive little benefit and have a high risk of early recurrence. Accordingly, a more selective, risk-adapted policy is warranted, and HDCT-ASCT should be restricted to carefully selected groups and integrated with novel approaches such as maintenance therapy, targeted agents or immunotherapy in order to consolidate the therapeutic benefit post-transplant.

Some key collaborative studies have also contributed to our knowledge about prognosis and therapeutic adaptation in Ewing sarcoma [3,15,16,17,18,19,20,21,22]. The Ahrens et al. CESS 86 study [17] also identified tumor volume as an important prognostic factor, and demonstrated that smaller tumors (<100 mL) and favourable histological response to induction chemotherapy associated closely with longer survival. These data helped to establish the risk-adapted designs subsequently introduced in Euro-EWING studies.

The Rizzoli Institute contributions Most of the evidence provided above comes from one large analysis by Bacci et al. found that poor histologic response, pelvic site of origin and large tumor size were independent predictors of worse survival, whereas younger age at diagnosis and location in extremity were associated with a favorable outcome [18]. These findings emphasise the relationship between initial tumour features and early treatment response in tailoring therapy.

Likewise, the Second UKCCSG/MRC Ewing’s Trial reported by Craft et al. showed that in older or disseminated patients, the addition of ifosfamide to the standard chemotherapy regimen significantly improved EFS, representing a development of the existing treatment. Younger or localized patients benefited even more; thus, ifosfamide is now considered a cornerstone of contemporary VIDE/VAI-based regimens [19].

So far, previous joint work of Jürgens et al. also accentuated the importance of multidisciplinary treatment. By adding chemotherapy to surgery and/or radiotherapy, 5-year survival rates more than 60% could be obtained for local disease, in contrast to the results with single-modality treatment [20]. This principle remains a fundamental one in the management of ES to this day.

In addition, McLean et al. that late relapses and treatment-related secondary tumours survived, even in long-term survivors, with approximately 12% of relapses over 5 years [21]. These results underscore the need for long-term follow-up and consideration of late-toxicity.

Finally, Paulussen and colleagues conducted the EICESS-92 study, which reported similar results for cyclophosphamide- and ifosfamide-based backbones in standard-risk disease. However, they observed superior 5-year EFS in high-risk patients when etoposide was added (41% vs. 54%, *p* = 0.03), leading to the development of the European VIDE/VAI/BuMel-based regimen [22].

Together, these observations support that prognosis in Ewing sarcoma is strongly correlated with tumor volume, histologic response, metastatic extent, and pattern of relapse. Treatment intensification (through HDCT-ASCT or systemic chemotherapy) should therefore be individualized based on each patient’s biological and clinical characteristics. Our findings of superior outcomes in younger patients and poorer survival with hepatic or pulmonary metastases corroborate this targeted, risk-adapted approach. Future clinical advances are likely to arise from combining targeted and immune-based therapies as part of consolidation or maintenance regimens, with the goal of improving survival while minimizing toxicity for patients with advanced or relapsed ES [17,18,19,20,21,22].

Novel national guidelines from the 2025 French FSG/NETSARC and Group OS groups reinforce these points, demonstrating a European consensus on the restricted use of HDCT-ASCT in cases of relapse or refractory disease. According to these guidelines, HDCT-ASCT cannot be considered a standard therapy because of inconsistent survival benefits and significant treatment-related toxicities. Rather, it may be appropriate only for a small subset of medically fit, chemosensitive patients managed in specialized multidisciplinary centres [23]. These findings are consistent with ours, in which HDCT–ASCT predominantly achieved temporary disease control in heavily pretreated or high-burden settings, with only marginal benefit observed in younger, chemosensitive patients. The guidelines also emphasize that future strategies should focus on combining targeted, immunologic, and maintenance approaches to modify outcomes, thereby echoing the translational implications of our findings. Collectively, these overlapping results suggest that the management of relapsed or refractory Ewing sarcoma is gradually shifting from empiric treatment intensification toward biologically driven precision therapy aimed at achieving durable responses without excessive toxicity [23].

## 5. Conclusions

In conclusion, while our study offers descriptive real-world insights into outcomes of locally advanced and metastatic ES patients undergoing HDCT-ASCT, these findings should be interpreted with caution given the small sample size and inherent limitations of a retrospective design. By integrating available clinicopathologic data with survival outcomes, we observed that age and metastatic distribution—particularly hepatic or pulmonary involvement—were associated with prognosis; however, these associations may not be generalizable beyond this selected cohort.

Although our results suggest that HDCT-ASCT provides limited ability to alter disease course in heavily pretreated or chemoresistant patients, these observations remain hypothesis-generating rather than definitive. The heterogeneity of our mostly adult patient population, combined with variability in prior treatments and disease biology, further underscores the need for prospective, adequately powered studies to clarify which subgroups may derive meaningful benefit from this approach.

Overall, our findings support a cautious, individualized use of HDCT-ASCT and highlight the importance of exploring consolidative or maintenance strategies—including immunotherapy and molecularly targeted approaches—to improve durability of disease control in this high-risk population.

## 6. Highlights

Despite improvements in multimodality treatment options, ES continues to represent a highly aggressive tumor with a poor prognosis following relapse.

In this single-center retrospective cohort of 46 heavily pretreated patients, the median post-transplant OS after HDCT-ASCT was 8 months, and PFS was 5 months.

**Figure 5:**
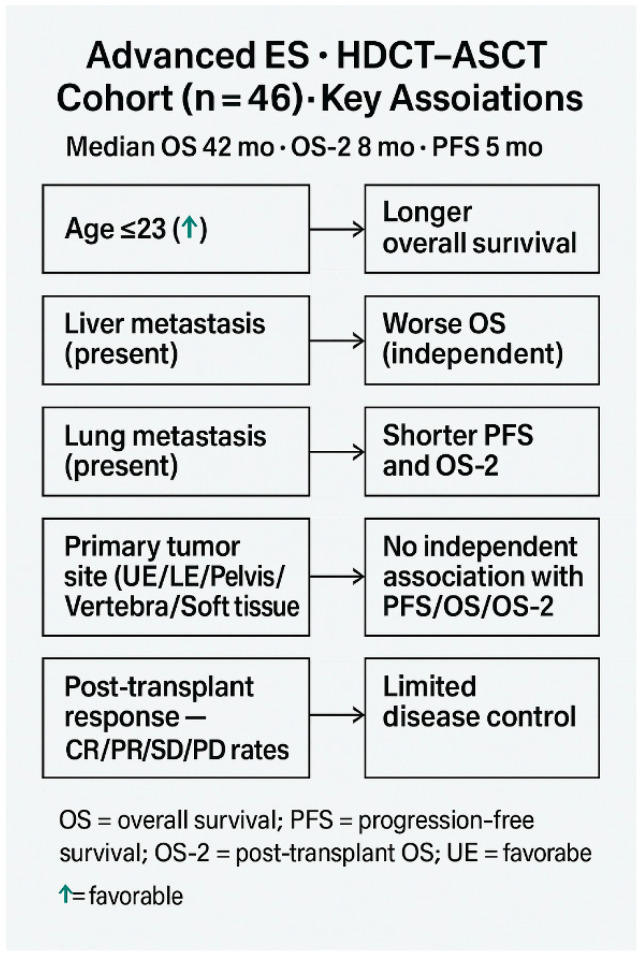
Summary of Prognostic Associations in Advanced Ewing Sarcoma Patients Receiving HDCT–ASCT.

Favorable prognostic parameters included younger age (≤23 years), chemosensitive disease, and absence of liver or lung metastases (Figure 5).

While HDCT-ASCT was generally well-tolerated, with a low treatment-related mortality of 2.1%, it provided only transient disease control in most patients.

These observations underscore the imperative to establish a risk-adapted, precision-based therapeutic algorithm in which HDCT-ASCT is reserved for appropriately selected, chemosensitive patients, in conjunction with targeted or immune therapies that may enhance post-transplant outcomes.

## 7. Limitations

This study is not without limitations that must be considered in interpreting these findings.

First, its retrospective and single-center design precludes the establishment of causality and limits generalizability. Selection bias may have been present due to referral patterns and institutional practices, which may not be representative of the general ES population.

Second, the sample size was relatively small (n = 46), potentially underpowered for subgroup analyses and unable to detect modest but clinically meaningful differences.

Third, heterogeneity in pretransplant strategies—such as different chemotherapy regimens, local control techniques, and timing of HDCT-ASCT—may have introduced confounding factors that impact survival outcomes.

Fourth, molecular and genetic data (e.g., *EWSR1* fusion type or *TP53* or *STAG2* mutations) were not uniformly collected, precluding analyses of biologic determinants of treatment response or resistance.

Fifth, there was no control group receiving conventional-dose salvage therapy with which to directly compare the relative efficacy of HDCT-ASCT.

Sixth, response and toxicity assessments were abstracted from medical records rather than a centralized, independent review, which may have introduced subjective reporting bias.

Finally, the follow-up period was adequate for primary survival analyses but may have been insufficient to capture late toxicities or delayed relapses.

Collectively, these restrictions underscore the importance of conducting further larger-scale, multicenter, prospective studies using standardized treatment protocols combined with comprehensive molecular profiling and longer follow-up duration, in order to validate these results and enhance patient selection for HDCT-ASCT.

## 8. Future Perspectives

Future research in advanced or relapsed Ewing sarcoma should aim to improve therapeutic effectiveness and achieve durable disease control through biology-driven, targeted strategies.

**Prospective Risk-Stratified Clinical Trials:** Integrating biological and clinical factors into risk assessment may help identify patients who benefit most from HDCT-ASCT. Minimal residual disease (MRD) monitoring and dynamic biomarkers could refine decisions regarding treatment intensity and timing. Recent multi-omics studies highlight substantial molecular heterogeneity driven by fusion-dependent transcriptional and epigenetic programs, suggesting that such signatures may enhance future risk-stratification frameworks [24,25].

**Combination of Targeted and Immunotherapeutic Treatments:** Combining targeted agents—such as PARP inhibitors, IGF-1R blockade, or CDK4/6 inhibitors—with immunotherapies including checkpoint inhibitors or cellular therapies may improve disease control, particularly in resistant cases. Multi-omics investigations identifying pathways such as CREB1–FGD4–LOXHD1 as contributors to metastatic progression provide a biological rationale for exploring such combinations [24].

**Development of Post-Transplant Maintenance Strategies:** Some patients may benefit from maintenance strategies using metronomic chemotherapy, immunomodulators, or low-dose targeted agents to prolong remission. Molecular biomarkers emerging from multi-omics platforms may help guide the selection of patients most likely to benefit from these approaches [25].

**Optimization of Multimodal Consolidation Therapy:** Further studies should assess combinations of HDCT with precision radiotherapy, surgery, or theranostic strategies to enhance both local and systemic control while minimizing toxicity. Insights from fusion-driven regulatory networks and metastasis-related signatures may support biologically informed consolidation approaches [24,25].

In summary, the treatment paradigm for Ewing sarcoma is shifting toward individualized, biology-guided multimodal care. HDCT-ASCT should be reserved for carefully selected, chemosensitive, and clinically fit patients, while future efforts prioritizing molecularly informed treatment algorithms and rational therapeutic combinations are strongly warranted.

## Figures and Tables

**Table 1:** Patient demographics and clinical characteristics.

Variable	n	%
**Sex**		
Male	32	69.6
Female	14	30.4
**Stage at Diagnosis**		
Locally advanced	36	78.3
Metastatic	10	21.7
**Tumor Localization**		
Upper Extremity	5	10.9
Lower Extremity	13	28.3
Vertebra	7	15.2
Pelvis	5	10.9
Soft Tissue	16	34.8
**Metastatic status**		
Lung	35	76.1
Liver	5	10.9
Bone	30	65.2
Brain	3	6.5
**Number of Therapy Lines Before HDC-ASCT**		
≤2	37	80.4
>2	9	19.6

**Table 2:** Hazard ratios and *p*-values by metastatic site for OS, OS-2, and PFS.

Metastasis Site	OS HR	OS *p*	OS-2 HR	OS-2 *p*	PFS HR	PFS *p*
**Lung**	1.175	0.71	2.672	0.025	6.037	0.016
**Liver**	5.411	0.006	1.683	0.351	1.896	0.261
**Bone**	1.226	0.587	0.861	0.688	0.705	0.413
**Brain**	2.602	0.142	1.353	0.628	0.717	0.665

**Table 3:** Treatment-Related Toxicities and Severity Grades Following HDCT–ASCT.

Toxicity	n	%
**Febrile neutropenia**	**46**	**100%**
Grade 3–4	46	100%
**Neutropenia**	**46**	**100%**
Grade 1–2	10	21.7%
Grade 3–4	36	78.3%
**Anemia**	**40**	**86.9%**
Grade 1–2	15	32.6%
Grade 3–4	25	54.3%
**Thrombocytopenia**	**46**	**100%**
Grade 1–2	-	-
Grade 3–4	46	100%
**Mucositis/stomatitis**	**30**	**65.2%**
Grade 1–2	26	56.5%
Grade 3–4	4	8.7%
**Vomiting**	**38**	**82.6%**
Grade 1–2	8	17.4%
Grade 3–4	30	65.2%
**Diarrhea**	**36**	**78.2%**
Grade 1–2	4	11.1%
Grade 3–4	32	88.9%
**Liver toxicity**	**10**	**21.7%**
Grade 1–2	10	21.6%
Grade 3–4	-	-
**Renal toxicity**	**8**	**17.3%**
Grade 1–2	8	17.3%
Grade 3–4	0	0
**Death**	**1**	**2.1%**

## Data Availability

This manuscript does not report data generation or analysis. Therefore, there are no datasets available for public access.
